# A systematic review evaluating the influence of incisional Negative Pressure Wound Therapy on scarring

**DOI:** 10.1111/wrr.12858

**Published:** 2020-08-21

**Authors:** Pieter R. Zwanenburg, Floyd W. Timmermans, Allard S. Timmer, Esther Middelkoop, Berend T. Tol, Oren Lapid, Miryam C. Obdeijn, Sarah L. Gans, Marja A. Boermeester

**Affiliations:** ^1^ Department of Surgery, Amsterdam Gastroenterology and Metabolism, Amsterdam Infection & Immunity, Amsterdam UMC University of Amsterdam Amsterdam The Netherlands; ^2^ Department of Plastic, Reconstructive & Hand Surgery Amsterdam Movement Sciences Research Institute, Amsterdam UMC, Vrije Universiteit Amsterdam Amsterdam The Netherlands; ^3^ Association of Dutch Burn Centers, Red Cross Hospital Beverwijk The Netherlands; ^4^ Department of Plastic, Reconstructive & Hand Surgery, Amsterdam UMC University of Amsterdam Amsterdam The Netherlands

## Abstract

Pathological scars can result in functional impairment, disfigurement, a psychological burden, itch, and even chronic pain. We conducted a systematic review to investigate the influence of incisional Negative Pressure Wound Therapy (iNPWT) on scarring. PubMed, EMBASE and CINAHL were searched for preclinical and clinical comparative studies that investigated the influence of iNPWT on scarring‐related outcomes. Individual studies were assessed using the OHAT Risk of Bias Rating Tool for Human and Animal studies. The body of evidence was rated using OHAT methodology. Six preclinical studies and nine clinical studies (377 patients) were identified. Preclinical studies suggested that iNPWT reduced lateral tension on incisions, increased wound strength, and reduced scar width upon histological assessment. Two clinical studies reported improved patient‐reported scar satisfaction as measured with the PSAS (1 year after surgery), POSAS, and a VAS (both 42, 90, and 180 days after surgery). Five clinical studies reported improved observer‐reported scar satisfaction as measured with the VSS, SBSES, OSAS, MSS, VAS, and POSAS (7, 15, 30, 42, 90, 180, and 365 days after surgery). Three clinical studies did not detect significant differences at any point in time (POSAS, VAS, and NRS). Because of imprecision concerns, a moderate level of evidence was identified using OHAT methodology. Preclinical as well as clinical evidence indicates a beneficial influence of iNPWT on scarring. Moderate level evidence indicates that iNPWT decreases scar width and improves patient and observer‐reported scar satisfaction.

## 1 INTRODUCTION

Each year, surgeons create over 200 million incisions.^1^ All these procedures are at risk of pathological scar formation. Pathological scars may result in disfigurement,^2^ chronic pain,^3^ itch,^4^ functional impairment,^5^ and a psychological burden.^6^ Pathological scars result in a need for additional treatments,^7^ including revisionary surgery.^8^ In the US alone, 170 000 scar revisions are performed each year.^9^ Incisional Negative Pressure Wound Therapy (iNPWT) is an increasingly applied treatment of surgical incisions, that has been shown to prevent postoperative wound complications such as surgical site infection and wound dehiscence.^10^, ^11^ Although it has been suggested that iNPWT may result in improved scar quality,^12^ the effect of iNPWT on scar formation still remains unclear. The aim of this paper is to systematically review preclinical and clinical studies that have investigated the influence of iNPWT on scar‐related outcomes. We hypothesize that iNPWT improves scar quality and reduces the formation of pathological scars.

## 2 MATERIALS AND METHODS

This systematic review and meta‐analysis was performed in agreement with the Preferred Reporting Items for Systematic Reviews and Meta‐Analyses (PRISMA) guidelines.^13^ A review protocol for this meta‐analysis was registered at PROSPERO (CRD42019122372). As this concerns a literature study, no ethical approval was required. A review protocol was registered at PROSPERO (CRD42019122372). A clinical librarian was consulted on the search strategy. PubMed, EMBASE, and CINAHL were searched from 2005 (the first paper on iNPWT was published in 2006) up to March 25th, 2019 (see Data S1 for the search strategy). The search strategy encompassed multiple MeSH terms, including “Negative‐Pressure Wound Therapy.” Titles and abstracts were screened by two independent reviewers (P.R.Z. and B.T.T.). Full texts of potentially eligible articles were reviewed based on predefined inclusion criteria by both reviewers.

Preclinical and clinical studies that investigated the influence of iNPWT on scar‐related outcomes were included. Outcomes of scar scales and quantitative measurements of wound/scar properties were regarded as relevant scar‐related outcomes. As excessive lateral tension around incisions increases the likelihood of pathological scar formation,^14^ we also included studies that performed finite element analyses in order to predict the influence of iNPWT on lateral incisional tension. Articles in languages other than English, German, and French were excluded, as were duplicates, congress abstracts, and articles without original data. References from included articles were also assessed for potential inclusion.

Two reviewers (P.R.Z. and F.W.T.) critically appraised each study using the Office of Health Assessment and Translation (OHAT) Risk of Bias Tool for Human and Animal Studies. We chose this tool because it allows for assessment of both animal as well as human clinical studies through a single framework. Discrepancies were resolved through discussion to reach a final risk of bias rating for each item, as guided by the instructions provided in the OHAT Handbook.^15^ Based on the design of an individual study, a number of items were rated to be at “definitely high,” “probably high,” “probably low,” or “definitely low” risk of bias. When studies did not report the necessary information “NR” (not reported) was recorded. One reviewer (P.R.Z.) extracted data in predefined evidence tables, that were checked subsequently by a second reviewer (F.W.T.). Disagreements were resolved through discussion until reach of consensus. Data collection included study characteristics and study outcomes such as results of finite element analyses, biomechanical tests, quantitative scar measurements, and patient and observer‐reported scar satisfaction assessments. We graded our confidence in the body of evidence using OHAT methodology,^15^ an adaptation of the Grading of Recommendations, Assessment, Development and Evaluation (GRADE) Working Group guidelines.

### 2.1 Statistical analysis

The extracted data was summarized in tables. A meta‐analysis was planned in case studies reported the same outcome. Reported values represent means from individual studies unless reported otherwise. Standard errors from individual studies are abbreviated as “SE,” whereas standard deviations are abbreviated as “SD.”

## 3 RESULTS

### 3.1 Systematic review

**FIGURE 1:**
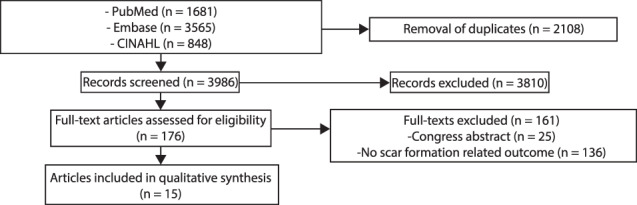
Systematic review flow diagram

The search strategy resulted in 6094 records. After removal of duplicates (n = 2108), 3986 records were screened by two independent reviewers (P.R.Z. and B.T.T.), and 3810 records were excluded based on title and abstract. A total of 176 full text articles was assessed for eligibility, after which 15 articles were included. An overview of the systematic review process is presented in Figure 1.

### 3.2 Study characteristics

We identified six *preclinical* articles, including four experimental animal studies^16^, ^17^, ^18^, ^19^ and two articles that combined the results of computer simulations (finite element analyses) with physical biomechanical benchmark testing.^12^, ^20^ Outcomes investigated by the animal studies included scar height,^18^, ^19^ scar width,^17^, ^18^, ^19^ wound strength during tensile testing,^16^, ^17^, ^18^ gene‐expression,^17^ color,^19^ overall appearance,^19^ histological assessment of collagen deposition^16^ and angiogenesis,^16^, ^19^ and laser Doppler imaging for perfusion assessment.^16^ The animal studies evaluated these outcome on either postoperative day (POD) 3, 4, 5, 7, 8, 21, or 40.^16^, ^17^, ^18^, ^19^

Nine *clinical* articles reported clinical patient and observer‐reported outcomes, among which five RCTs,^21^, ^22^, ^23^, ^24^, ^25^ three prospective comparative studies,^26^, ^27^, ^28^ and one retrospective comparative study.^29^ Clinical studies reported results after abdominoplasty,^29^ circumferential thigh lift,^26^ oncological breast surgery,^27^ breast tissue expansion,^21^ laparotomy,^25^, ^28^ vascular groin surgery,^22^ reduction mammoplasty,^23^ and coronary artery bypass grafting.^24^ Scar scales used by the clinical studies involved the Vancouver Scar Scale (VSS),^22^, ^29^ Stony Brook Scar Evaluation Scale (SBSES),^22^, ^26^ Observer Scar Assessment Scale (OSAS),^27^ Patient Scar Assessment Scale (PSAS),^22^, ^27^ Manchester Scar Scale (MSS),^27^ Body Image Scale (BIS),^27^ Visual Analog Scale (VAS),^21^, ^22^, ^23^, ^28^ Patient and Observer Scar Assessment Scale (POSAS).^23^, ^25^, ^28^ Clinical studies also performed scar width measurement,^21^, ^22^ immunohistochemistry (of tissue resected at tissue expander replacement surgery),^21^ scanning acoustic microscopy (a method used for assessment of tissue elasticity),^21^ measurement of scar viscoelasticity,^23^ skin water content,^23^ and transepidermal water loss (TEWL),^23^ and clinical assessment of hypertrophic scar formation events.^24^ Timing of clinical evaluations varied from POD 7 up to a maximum of 1194 days after surgery.^21^, ^22^, ^23^, ^24^, ^25^, ^26^, ^27^, ^28^

**FIGURE 2:**
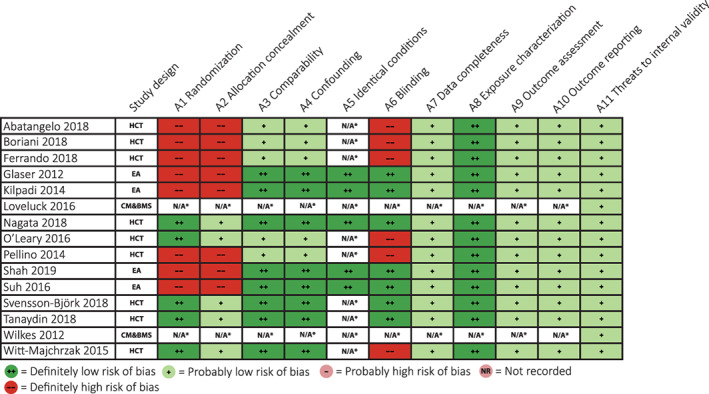
Office of health assessment and translation risk of bias tool assessment [Color figure can be viewed at wileyonlinelibrary.com]

OHAT Risk of Bias Tool scorings of the included studies are provided in Figure 2. Nine of fifteen studies reported involvement of industry funding.^12^, ^16^, ^17^, ^18^, ^19^, ^20^, ^23^, ^25^, ^28^

### 3.3 Preclinical studies: Finite element analyses

Two studies aimed to assess the effects of iNPWT on mechanical stress applied to incisional tissue by use of computer models, that is, finite element analyses (FEAs).^12^, ^20^ Wilkes et al used two FEA models with −125 mmHg of subatmospheric pressure; one concerning an incision with a subcutaneous void, and another model that incorporated fascial separation.^12^ In the first model, lateral tension at the skin level was reduced from 2.2 to 2.5 kPa, to 0.9 to 1.2 kPa (about 50%). In the second model, iNPWT significantly decreased the amount of lateral stress in epidermis (28.05, SE 1.98–14.82 kPa, SE 0.58 kPa, *P* = .235) but increased lateral stress at dermis level (14.50, SE 0.08–15.34, SE 0.15, *P* = .0407). Shear stress was reduced in epidermis (3.67, SE 0.14–0.12 kPa, SE 0.11 kPa, *P* = .0029), dermis (2.16, SE 0.20–0.38 kPa, SE 0.26 kPa, *P* = .0327), and fat (1.08, SE 0.27–0.04 kPa, SE 0.01 kPa, *P* < .0001).^12^ Loveluck et al used a singular computer model of an incision through skin, fat and muscle, where they applied −40 to −80 mmHg of subatmospheric pressure.^20^ In this model, the force on individual sutures was reduced from 1.31 to 0.56 N with −40 mmHg of subatmospheric pressure (a reduction of 57%), and from 1.31 to 0.40 N with −80 mmHg of subatmospheric pressure (a reduction of 69%).^20^

### 3.4 Preclinical studies: Biomechanical testing of incisions treated with iNPWT

Three porcine animal studies investigated the biomechanical properties of incisions treated with iNPWT compared with standard surgical dressings (SSDs).^16^, ^17^, ^18^ Glaser et al reported that incisional wounds treated with either 3 or 5 days of iNPWT displayed a higher failure load compared to wounds treated with SSDs (16.5, SD 14.6 vs 4.9 ± 4.0 N), meaning that iNPWT‐treated wounds could absorb more energy (8.0 ± 9.0 vs 26.9 ± 23.0 mJ) and withstand a larger amount of ultimate stress (62 ± 53 vs 204 ± 118 N/mm^2^) on POD 3 or 5. This study reported the results of 3 and 5 days of iNPWT as one group.^18^ Suh et al reported increased tensile strength after 7 days of iNPWT on POD 7 (24.6 vs 18.26 N, *P* < .05) and POD 21 (61.67 vs 50.05 N, *P* < .05) compared to SSDs.^16^ Kilpadi et al reported that 5 days of iNPWT resulted in a significantly increased amount of energy required for disruption of healed incisions compared to SSDs on POD 40 (0.21, SE 0.04 N/mm^2^ vs 0.15, SE 0.02 N/mm^2^, *P* = .0373).^17^

### 3.5 Preclinical studies: Quantitative scar measurements and scar scale assessment

Two studies performed quantitative scar measurements.^17^, ^18^ Kilpadi et al measured scar width in deep and superficial dermis of incisions treated with either iNPWT or SSDs on POD 40 by using photographs of porcine histological specimens. They reported a decreased deep dermal scar width (1.313, SE 138 vs 1.000, SE 131 μm, *P* = .0215), whereas upper dermal scar width did not differ (605, SE 72 vs 645 μm, SE 78 μm, *P* > .1).^17^ Glaser et al measured scar width of histological specimens taken at either POD 3 or 5 (results were reported as one group), and reported that iNPWT resulted in a non‐significantly decreased scar width (236 vs 93 μm, *P* = .2).^18^ Scar height was also assessed by using photographs taken after dressing removal, subjected to blinded assessment by a single observer. Five of eight incisions treated with SSDs received a scar height grade of 1 (0 = normal, 1 = <2 mm), and three incisions received a grade of 0. Incisions treated with iNPWT all received a scar height grade of 0, resulting in a significantly decreased scar height (*P* = .026).^18^ In their porcine model, Shah et al performed SBSES scar assessment of photographs of closed surgical wounds taken after 8 days of iNPWT, SDDs, or inactive iNPWT. Scar width, height, color, and overall appearance were assessed by three blinded observers.^19^ Incisional NPWT was reported to result in improved scar width compared to SSDs (0.94, SE 0.04 vs 0.47, SE 0.09, *P* < .0001), improved scar height compared to inactive iNPWT (0.97, SE 0.03 vs 0.69, SE 0.08), improved scar color compared to SSDs (0.91, SE 0.05 vs 0.60, SE 0.09, *P* = .013), and improved overall appearance compared to SSDs (0.94, SE 0.04 vs 0.53, SE 0.09) and inactive iNPWT (0.94, SE 0.04 vs 0.66, SE 0.09).^19^ A summary of the preclinical studies is presented in Table 1.

**TABLE 1:** Summary of preclinical studies

Reference	Model	Treatment	Methods	Sample size	Timing of Evaluation	Results
Glaser 2012^18^	Porcine	iNPWT (3 or 5 days, but reported as one group) vs SSDs in two spinal incisions. Used Prevena dressings (manufactured by 3M/KCI).	Biomechanical testing (wound strength measurement), histological evaluation of excised wounds, and modified VSS assessment on POD 3 and 5	N = 8	POD 3 or 5 but reported as one group	VSS: decreased scar height with iNPWT*
						Biomechanical testing: increased failure load (4.9, SD 4.0 vs 16.5, SD 14.6 N), energy absorption (8.0, SD 9.0 vs 26.9, SD 23.0 mJ), and ultimate stress capacity (62, SD 53 vs 204, SD 118 N/mm^2^)
						Histology: decreased scar width (236 vs 93 μm)
Kilpadi 2014^17^	Porcine	iNPWT vs SSDs of four pairs of dorsal incisions. Used Prevena dressings (manufactured by 3M/KCI).	Biomechanical testing, histological evaluation of excised wounds on day 40 (n = 12 pairs/group), biopsy for gene‐expression analysis on days 5 (n = 6 pairs/group), 20 (n = 6 pairs/group), and 40 (n = 12 pairs/group)	N = 6	POD 40	Histology: More narrow deep dermal scar (1000, SE 131 vs 1313, SE 138 μm), similar upper dermal scar width (605, SE 72 μm vs 645, SE 78 μm)
						Improved mechanical properties* (strain energy density: 0.21, SE 0.04 N/mm^2^ vs 0.15, SE 0.02 N/mm^2^, peak strain: 0.23, SE 0.02 vs 0.18, SE 0.01)
						Less upregulation of genes associated with inflammation, hypoxia, retardation of reepithelialization, impaired wound healing and scarring with iNPWT
Shah 2019^19^	Porcine	iNPWT vs inactive iNPWT or a SSD of 3 dorsal incisions and 1 untreated skin area. Used GranuFoam dressings (manufactured by 3M/KCI).	Blinded SBSES, immunohistochemical VEGF assessment, ELISA VEGF assessment	N = 9	POD 8	SBSES: improved scar width compared to SSDs* (0.94, SE 0.04 vs 0.47, SE 0.09,), improved scar height compared to inactive iNPWT (0.97, SE 0.03 vs 0.69, SE 0.08), improved color compared to SSDs* (0.91, SE 0.05 vs 0.60, SE 0.09), improved overall appearance compared to SSDs* (0.94, SE 0.04 vs 0.53, SE 0.09) and inactive iNPWT* (0.94, SE 0.04 vs 0.66, SE 0.09). Improved total SBSES score (3.75, SE 0.09) compared to SSDs* (2.38, SE 0.26) and inactive iNPWT* (2.78, SE 0.22)
						Immunohistochemistry: Increased VEGF and Factor VIII staining
						ELISA: 2.8% vs 1% VEGF*
Suh 2016^16^	Porcine	iNPWT vs SSDs with 2 spinal incisions. Used CuraVAC dressings (manufactured by Daewoong Pharmaceutical Co., Ltd)	Laser Doppler imaging perfusion assessment on days 4, 7, and 21, tensile strength and histology assessment on days 7 and 21	N = 6	4, 7, and 21	Increased tensile strength at POD 7 (24.6 vs 18.26 N) and POD 21* (61.67 vs 50.05 N)
						Laser Doppler Imaging: Improved perfusion on days 4*, 7*, and 21*
						Histology: Improved collagen deposition and angiogenesis
Wilkes 2012^12^	Computer model	Prevena model (manufactured by 3M/KCI)	FEA1 (computer model of an incision with a subcutaneous void), FEA2 (computer model of incision with fascial separation), bench testing (synthetic skin model)	NA	NA	FEA1: reduced lateral tension at skin level (from 2.2‐2.5 to 0.9‐1.2 kPa, about 50%)
						FEA2: decreased lateral stress in epidermis* (28.05, SE 1.98–14.82 kPa, SE 0.58 kPa) and dermis (14.50, SE 0.08–15.34, SE 0.15), decreased shear stress in epidermis* (3.67, SE 0.14–0.12 kPa, SE 0.11 kPa), dermis* (2.16, SE 0.20–0.38 kPa, SE 0.26 kPa), and fat* (1.08, SE 0.27–0.04 kPa, SE 0.01 kPa)
						Biomechanical testing: More force needed for disruption of incisions treated with iNPWT* (from 61.7, SE 0.3–92.9 N, SE 2.6 N, a 51% increase)
Loveluck 2016^20^	Computer model	PICO (Manufactured by Smith & Nephew)	FEA (computer model), bench testing (synthetic skin model)	NA	NA	FEA: Reduced force on individual sutures from 1.31 to 0.56 N (43%) and from 1.31 to 0.40 N (31%) with −40 and − 80 mmHg of subatmospheric pressure
						Biomechanical testing: −80 mmHg resulted in a 55% increase of the amount of force required for incisional deformation

Abbreviations: ELISA, enzyme‐linked immunosorbent assay; FEA, finite element analysis; iNPWT, incisional Negative Pressure Wound Therapy; POD, postoperative day; SBSES, Stony Brook Scar Evaluation Scale; SSD, standard surgical dressing; VEGF, vascular endothelial growth factor; VSS, Vancouver Scar Scale.

^*^ Statistically significant result (*P* < .05).

### 3.6 Clinical studies: Quantitative scar measurements

Nagata et al measured the scar width of 13 incisions of women undergoing tissue expansion for breast reconstruction, where they randomized half of the incisional wound to iNWPT, and the other half to film dressing treatment. All patients received a minimum of 42 days of treatment (average 58.5, range 42‐81 days). Scar width was measured by using photographic image analysis after 6 months, and demonstrated a decreased scar width to be associated with iNPWT (2.92 vs 4.75 mm, *P* = .0015).^21^ Although the paper did not provide numeric values, scars treated with iNPWT were also reported to be softer as measured with a scanning acoustic microscope (a technique used to measure tissue elasticity).^21^ Tanaydin et al measured scar viscoelasticity, skin water content, and transepidermal water loss on POD 42, 90, 180, and 365 of 32 women undergoing bilateral breast reduction mammoplasty. Through randomization, each side received either iNPWT or fixation strips. They reported that skin viscoelasticity, transepidermal water loss, and hydration measurements did not show significant improvement with iNPWT (the paper only provided graphs, numeric values were not provided).^23^

### 3.7 Clinical studies: Patient‐reported scar evaluation

Four clinical studies (142 patients) assessed patient‐reported scar satisfaction.^22^, ^23^, ^27^, ^28^ These studies included two RCTs^22^, ^23^ and two prospective comparative studies.^27^, ^28^ In the observational study by Ferrando et al, 47 patients undergoing oncological breast surgery (25 patients received iNPWT, 22 patients received SSDs) were assessed with the BIS and PSAS after 1 year of follow‐up. Although BIS results did not differ between groups (*P* = .58), iNPWT patients reported an improved PSAS score (*P* = .002).^27^ In their randomized study of 32 bilateral breast reduction mammoplasty patients, Tanaydin et al found that patients reported significantly improved scar satisfaction with iNPWT as compared to their contralateral breast treated with SSDs on POD 42, 90, and 180, as measured by the POSAS and a VAS, whereas the effect became non‐significant 1 year after surgery.^23^

The comparative study of Pellino et al assessed patient‐reported scar satisfaction on POD 90 after abdominal surgery for Crohn's disease among 30 patients, by using the POSAS and a VAS. No significant differences were detected in any domain of the POSAS (pain, itchiness, color, stiffness, thickness, irregularity, or total score), nor did they detect a difference in VAS‐measured scar appearance (6.9, SD 2.5 vs 7.1, SD 2.1, *P* = .795).^28^ Likewise, Svensson‐Björk et al reported they used PSAS assessment of 33 patients with bilateral inguinal incisions randomized to either iNPWT or SSDs, and did not detect significant differences in any domain or total score of the PSAS (pain, itching, color, stiffness, thickness, irregularity, overall satisfaction) at a median of 808 days after surgery (range 394‐1194).^22^

### 3.8 Clinical studies: Observer‐reported scar evaluation

Nine clinical studies provided observer‐reported scar satisfaction results of 377 patients.^21^, ^22^, ^23^, ^24^, ^25^, ^26^, ^27^, ^28^, ^29^ These studies included five RCTs,^21^, ^22^, ^23^, ^24^, ^25^ three prospective comparative studies,^26^, ^27^, ^28^ and one retrospective study.^29^ Five studies (180 patients) reported significant improvement of observer‐reported scar quality as measured with the VSS,^29^ SBSES,^26^ OSAS,^27^ MSS^27^), VAS,^21^, ^23^ and POSAS.^23^ Significant improvement was reported on POD 7,^26^ 15,^26^ 30,^26^ 42,^23^ 90,^23^, ^26^, ^29^ 180,^23^ and 365.^26^, ^27^ Nagata et al reported improved scar quality 6 months after >42 days of iNPWT.^21^ Three studies (113 patients) did not report significant improvement with iNPWT as measured by POSAS,^22^, ^25^, ^28^ VAS,^25^, ^28^ and a NRS^22^ on POD 30,^24^ 90,^27^ and 808 (median POD of evaluation).^22^ Witt‐Majchrzak et al reported a non‐significant decrease of hypertrophic scarring with use of iNPWT (from 7/40 to 3/40, *P* = .1615). Only two studies (63 patients in total) reported the results of individual domains of the scar scales used in their studies.^22^, ^28^ Both studies did not detect significant differences in any domain (pain, itch, color, stiffness, thickness, or irregularity).^22^, ^28^ A summary of the clinical studies is presented in Table 2.

**TABLE 2:** Summary of clinical studies

Reference	Design	Control	n, total	Procedure/incision type	iNPWT technique	Control dressings	Outcome measures	Timing of evaluation	Results
Abatangelo 2018^29^	RCS	Inter‐patient	11	Post‐bariatric abdominoplasty	Prevena, manufactured by 3M/KCI (−125 mmHg for eight consecutive days)	“Non‐adherent control dressings (Inadine™, Systagenix, San Antonio, Texas)”	VSS	POD 90	iNPWT improved scar quality* (mean VSS: 2, SD 1 vs 6.5, SD 1).
Boriani 2018^26^	PCS	Inter‐patient	91	Circumferential thigh lift	Prevena, manufactured by 3M/KCI (−125 mmHg for seven consecutive days)	“Standard surgical dressings”	SBSES	POD 7, 15, 30, and 365	iNPWT improved SBSES score at 7*, 15*, 30*, and 365* days.
Ferrando 2018^27^	PCS	Inter‐patient	37	Oncological breast surgery	Prevena, manufactured by 3M/KCI (−125 mmHg for 7 consecutive days)	“Steri‐strip closure for 14 days, changed after 7 days”	OSAS and MSS (plastic surgeon), BIS and PSAS (patient)	POD 365	iNPWT improved PSAS* 11 (6‐18) vs 20 (14‐34); OSAS* 7 (6‐13) vs 24 (17‐29); MSS* 7 (5‐12) vs 12 (9‐15); BIS 6 (1‐14) vs 6 (3‐14.5)
Nagata 2018^21^	RCT	Intra‐patient	13	Breast tissue expansion	iNPWT, without use of a specific commercially available product (−125 mmHg for ≥6 weeks with weekly dressing changes) as half‐side comparison	“Film dressings changed once a week (Airwall; Kyowa, Osaka, Japan)”	VAS, scar width measurement, immunohistochemical staining, scanning acoustic microscopy	POD 183 (=6 months)	Lower VAS with iNPWT* (2.38 vs 3.95, −1.57), narrower scar with iNPWT* (2.92 mm vs 4.75 mm)
O'Leary 2017^25^	RCT	Inter‐patient	50	Laparotomy	PICO, manufactured by Smith & Nephew (−80 mmHg for four consecutive days)	“Transparent waterproof dressings (Smith & Nephew)”	POSAS	POD 30	No difference (32.6 vs 31.7, *P* < .89)
Pellino 2014^28^	PCS	Inter‐patient	30	Laparotomy (for Crohn)	PICO, manufactured by Smith & Nephew (−80 mmHg “for seven consecutive days or when a complication occurred”)	“Basic wound contact absorbent dressings”	POSAS, VAS	POD 90	No significant differences for any comparison
Svensson‐Björk 2018^22^	RCT	Inter‐patient	33	Vascular groin surgery	PICO, manufactured by Smith & Nephew (−80 mmHg for seven consecutive days)	“Sterile, waterproof dressings with a central absorbent pad (ViTri Medical, Stockholm, Sweden)”	PSAS, POSAS, OSAS, SBSES, VSS, VAS, scar width measurement	POD 808 (median, range 394‐1194)	No significant differences between iNPWT and SSDs
Tanaydin 2018^23^	RCT	Intra‐patient	32	Reduction mammoplasty	PICO, manufactured by Smith & Nephew (−80 mmHg for 14 days, with a dressing change at 7 days)	“Steri‐Strip dressings (3 M, St. Paul, Minnesota)”	POSAS, VAS, scar viscoelasticity, skin water content, TEWL	POD 42, 90, 180, and 365	Improved POSAS at 42* and 90* days with iNPWT (values NR), improved VAS at 42*, 90*, and 180* days with iNPWT (values NR). No significant differences in scar viscoelasticity, skin water content and TEWL (values NR).
Witt‐Majchrzak 2015^24^	RCT	Inter‐patient	80	CABG	PICO, manufactured by Smith & Nephew (−80 mmHg for up to 6 days, with a dressing change on day 2 or day 3)	“Conventional wound dressings”	Clinical assessment of hypertrophic scar formation, serous vesicle formation and marginal skin necrosis	POD 42	Decreased incidence of hypertrophic scar formation (iNPWT 3/40 (7.7%) vs SSDs 7/40 (18.4%), less marginal skin necrosis* (iNPWT 0/40 vs SSDs 12/40, 30%)

Abbreviations: BIS, body image scale; CABG, coronary artery bypass grafting; iNPWT, incisional Negative Pressure Wound Therapy; MSS, Manchester scar scale; NR, not reported; OSAS, observer scar assessment scale; PCS, prospective comparative study; POD, postoperative day; POSAS, patient and observer scar scale; PSAS, patient scar assessment scale; RCS, retrospective comparative study; RCT, randomized controlled trial; SBSES, Stony Brook scar scale; SSDs, standard surgical dressings; TEWL, transepidermal water loss; VAS, visual analogue scale; VSS, Vancouver Scar Scale.

^*^ Statistically significant result (*P* < .05).

### 3.9 Safety and iNPWT‐related adverse events

No adverse reactions related to iNPWT were reported by any of the included studies.

### 3.10 Level of evidence

Because the exposure (iNPWT) was experimentally controlled, occurred prior to the development of the outcome, and the outcome was assessed on the individual level, the body of evidence received an initial “high confidence” rating. Because most studies had small sample sizes, we downgraded the level of evidence because of imprecision concerns. Ultimately, we identified a “moderate” level of evidence. An overview of the rating process according to the OHAT approach is presented in Table 3.

**TABLE 3:** Rating of body of evidence according to the OHAT approach

Assessment	Initial rating	Risk of bias	Unexplained inconsistency	Directness and applicability	Imprecision	Publication bias	Magnitude	Dose‐ response relationship	Residual confounding	Cross‐species/population/study consistency	Final rating
	High	No downgrade	No downgrade	No downgrade	Downgrade	Not detected	No upgrade	No upgrade	No upgrade	No upgrade	Moderate
Explanation	*Initial rating*: As the exposure was experimentally controlled, occurred prior to the development of the outcome, and the outcome was assessed on the individual level with appropriate comparisons, the body of evidence received a high initial rating. *Risk of bias*: As the body of evidence also included randomized studies with blinded outcome assessment, we did not downgrade because of risk of bias. *Unexplained inconsistency*: Although not all studies indicated a significant benefit of iNPWT on scar formation, we consider it unclear whether this is the result of the limited sample sizes, a lack of an effect, or other causes, and therefore did not downgrade because of unexplained inconsistency. *Directness and applicability*: All studies their methodology was also aimed at addressing scar‐related outcomes, we did not downgrade because of indirectness. *Imprecision*: We considered all studies to have limited sample sizes (all less than 100 participants). In order to detect subtle differences in scar formation at 1 year postoperatively, we presume large sample sizes to be required. As a result, we downgraded because of imprecision concerns. *Publication bias*: Although a considerable amount of studies reported they had received industry support, we did not detect evident signs of publication bias. *Magnitude*: Because of the limited amount of evidence we did not upgrade because of effect magnitude. *Dose‐response relationship*: The included studies did not present data to evidently suggest the presence of a dose‐response relationship. *Residual confounding*: Some studies excluded patients that developed wound complications. This could be considered residual confounding. Yet, because of the limited amount of identified evidence, we did not upgrade because of residual confounding. *Study consistency*: The body of evidence exhibits incongruences between studies, with some clinical studies reporting improvements, while other studies report an absence of any effect of iNPWT. We therefore did not consider an upgrade to be justified. *Final rating*: Moderate										

Abbreviation: iNPWT, incisional Negative Pressure Wound Therapy.

## 4 DISCUSSION

This study provides an overview of the literature regarding the influence of iNPWT on scar formation (six preclinical studies,^12^, ^16^, ^17^, ^18^, ^19^, ^20^ nine clinical studies with a total of 377 patients^21^, ^22^, ^23^, ^24^, ^25^, ^26^, ^27^, ^28^, ^29^). Overall, we identified moderate level evidence that indicates that iNPWT improves scarring.

Preclinical studies indicated that iNPWT reduced incisional tension,^12^, ^20^ increased the amount of force needed to disrupt incisions,^16^, ^17^, ^18^ and decreased scar/granulation tissue width as judged by photographic assessment^19^ and blinded histological specimen measurement.^17^, ^18^ Nevertheless, all preclinical evaluations were performed within 40 days after surgery; long‐term outcomes would be more appropriate but less feasible in experimental settings.

Clinical scar width measurements 6 months after surgery indicated a reduced scar width after more than 6 weeks of iNPWT (13 patients).^21^ Two clinical studies reported that iNPWT resulted in a significant improvement of *patient* scar satisfaction as graded with the PSAS (1 year after surgery, 37 patients),^27^ POSAS and VAS (up to 180 days after surgery, 32 patients),^23^ where two other studies did not detect significant differences as measured with POSAS and VAS (POD 90, 30 patients^28^), and PSAS (median POD 808, 33 patients).^22^ Five studies reported that iNPWT resulted in improved *observer*‐reported scar satisfaction compared to standard surgical dressings as measured with various scar scales between 90 and 365 postoperative days in a total of 184 patients.^21^, ^23^, ^26^, ^27^, ^29^ Nevertheless, three studies did not detect significant *observer*‐reported improvement after iNPWT as measured with various scar scales between 30 and 808 postoperative days in a total of 113 patients.^22^, ^25^, ^28^ One study reported a non‐significant decrease of hypertrophic scarring events on POD 42.^24^ Finally, an overall moderate level of evidence was identified because the level of evidence was downgraded as a consequence of imprecision concerns.

Excessive lateral tension is generally considered an important causal factor in pathological scar formation,^14^ and other treatments aimed at reduction of incisional tension (eg, by offloading incisions by use of adhesive strips) have previously confirmed favorable results.^30^, ^31^ As iNPWT reduces lateral tension in similar fashion,^12^, ^20^ a beneficial effect of iNPWT appears comprehensible. Indeed, most studies of this systematic review seem to confirm a positive influence of iNPWT on pathological scar formation, as demonstrated by both subjective patient and observer evaluations and objective quantitative measurements such as scar width.

In addition, postoperative wound complications such as wound dehiscence, surgical site infection, or skin necrosis are also notorious causes of pathological scar formation.^32^ As several meta‐analyses indicate that iNPWT reduces the incidence of these postoperative wound complications,^10^, ^33^ there is a substantial amount of indirect evidence to suggest a beneficial effect of iNPWT on scar quality in general.

This systematic review has several limitations. One finding of our systematic review is that differences between groups seem to become increasingly difficult to detect with time, as many small studies could not detect an effect after 1 year of surgery, whereas the effect remained intact in the larger study.^26^ Yet, most studies had limited sample sizes and length of follow‐up, and scar‐related outcomes were a secondary outcome in most of the identfied studies. We did not identify any study that performed an a priori sample size calcution for a scar formation‐related outcome. A considerable number of studies reported they had received industry funding (9 of 15 studies).^12^, ^16^, ^17^, ^18^, ^19^, ^20^, ^23^, ^25^, ^28^ When considering the patient‐specific nature of scar formation, another methodological limitation of the present literature is the limited evidence available from intra‐patient controlled studies (only two small studies were identified).^20^, ^22^ Quantitative clinical scar measurements were only available for a limited amount of patients. A meaningful meta‐analysis could not be performed because of methodological heterogeneity. Because of the scarcity of reports that provided outcomes for specific scar scale domains, a meaningful analysis of distinct domains (such as pain, itch, or scar appearance) was also precluded.

Although a beneficial effect of iNPWT on scar quality seems to be present, the evidence is of moderate level because it suffers from imprecision due to insufficient number of patients. Moreover, only one study addresses cost‐effectiveness of iNPWT. Although Abatangelo et al report that iNPWT reduces the total costs for management of local wound complications ($750 vs $1066), their study has a limited sample size of only 11 patients. Ideally, an adequately powered intra‐patient controlled RCT with adequate length of follow‐up and cost‐effectiveness analysis should be performed in order to confirm or refute the results of this systematic review.

The evidence summarized in this review suggests that iNPWT seems to reduce pathological scar formation and improve scar quality. Incisional NPWT also seems to reduce the risk of other postoperative wound complications,^10^ and may be less labor intensive than conventional postoperative wound care. No iNPWT‐related adverse events were reported by any study included in this review. This suggests that iNPWT represents a sensible postoperative wound care strategy to be considered by clinicians. The finding that Nagata et al detected significant scar improvement after ≥6 weeks of iNPWT despite their limited sample size of only 13 patients suggests that prolonged iNPWT duration (more than 6 weeks) may have an especially beneficial influence on scar quality.

Both preclinical studies and clinical studies suggest a beneficial effect of iNPWT on scar quality. Moderate level evidence indicates that iNPWT results in smaller scars, and improves patient and observer‐reported scar satisfaction.

## CONFLICT OF INTEREST

M.A. Boermeester reports institutional grants from J&J/Ethicon, Acelity/KCI, Allergan/LifeCell, Ipsen, Mylan, and is advisory board member of J&J/Ethicon, 3M/KCI, Bard. M.A. Boermeester is also speaker and/or instructor for 3M/KCI, Allergan/LifeCell, Bard Davol BD, Johnson&Johnson/Ethicon, Gore, and Smith & Nephew. E. Middelkoop reports institutional grants from Elastagen Pty Ltd, Micreos BV, Cutiss AG for research outside of the submitted work. O. Lapid is a speaker for Smith & Nephew. P.R. Zwanenburg is a speaker for GD Medical Pharma BV and Hospithera NV. The other authors do not declare any conflicts of interest. Please note that 3M, KCI, Smith & Nephew, GD Medical Pharma, and Hospithera NV are companies involved in the production or distribution of iNPWT technology.

## AUTHOR CONTRIBUTIONS

All authors contributed to the final design of the manuscript, interpreted data, and helped draft the final manuscript, and revised it critically in equal measure as a group effort.

All authors approve the final version to be published and agree to be accountable for all aspects of the work related to its accuracy and integrity.

Pieter R. Zwanenburg also formulated the first concept for this manuscript and its methodology, constructed the search strategy together with a clinical librarian, performed the systematic review, helped with data extraction, risk of bias assessment and OHAT assessment of the body of evidence, and helped with the construction of the tables, created the figures and illustrations, and devised the initial outlines of the manuscript. Allard S. Timmer and Berend T. Tol helped with this process.

Floyd W. Timmermans also performed the risk of bias assessment and OHAT assessment of the body of evidence.

Oren Lapid and Miryam C. Obdeijn also provided their clinical perspective as plastic surgeons.

Professor Marja A. Boermeester and Professor Esther Middelkoop also acted as study supervisors.

**Abbreviations**
BIS: body image scale
CINAHL: cumulative Index of Nursing and Allied Health Literature
Cm: centimeter
EMBASE: Excerpta Medica dataBASE
FEA: finite element analysis
iNPWT: incisional Negative Pressure Wound Therapy
kPA: kilopascal
mmHg: millimeters of Mercury
MSS: Manchester Scar Scale
N: Newton
NRS: Numeric Rating Scale
OHAT: Office of Health and Technology
POD: postoperative day
POSAS: Patient and Observer Scar Assessment Scale
PSAS: Patient Scar Assessment Scale
SBSES: Stony Brook Scar Evaluation Scale
SSDs: Standard Surgical Dressings
TEWL: transepidermal water loss
VAS: Visual Analog Scale
VSS: Vancouver Scar Scale
μm: micrometer

## Supporting information


**Data S1** Supporting Information
