# Supplementary material for: A systematic review evaluating the influence of incisional Negative Pressure Wound Therapy on scarring
Source: Wound Repair Regen. 2020 Aug 21;29(1):8–19. doi: 10.1111/wrr.12858 (PMC7891404; doi:10.1111/wrr.12858)
Supplement: Supplementary file 1 — Data S1 Supporting Information [file WRR-29-8-s001.pdf]

SUPPLEMENTARY FILE 1 – THE SEARCH STRATEGY

From 2005 up to March 25th, 2019.

PubMed:

("Negative-Pressure Wound Therapy"[Mesh] OR negative pressure*[tiab] OR NPWT[tiab] OR NPT[tiab] OR vacuum assisted closure*[tiab] OR VAC[tiab] OR V.A.C.[tiab] OR TNP[tiab] OR (topical[tiab] AND subatmospheric pressure*[tiab]) OR TASAP[tiab] OR microdeformational wound therap*[tiab] OR vacuum therap*[tiab] OR closed incision management[tiab] OR surgical incision management[tiab] OR incisional management system*[tiab] OR prevena[tiab])

AND

("Surgical Wound Infection"[Mesh] OR "Surgical Wound Dehiscence"[Mesh] OR "Seroma"[Mesh] OR "Hematoma"[Mesh] OR "Necrosis"[Mesh] OR surgical site infection*[tiab] OR surgical wound infection*[tiab] OR surgical wound*[tiab] OR surgical incision*[tiab] OR surgical site complication*[tiab] OR surgical site occurrence*[tiab] OR surgical site event*[tiab] OR incision*[tiab] OR prophylactic*[tiab] OR seroma*[tiab] OR necrosis[tiab] OR cellulitis[tiab] OR dehiscence*[tiab] OR hematoma*[tiab])

Embase (Ovid):

1 vacuum assisted closure/ or (negative pressure or NPWT or NPT or vacuum assisted closure* or VAC or TNP or TASAP or microdeformational wound therap* or vacuum therap* or closed incision management or surgical incision management or incisional management system* or prevena).ti,ab,kw. or (topical adj6 subatmospheric pressure*).ti,ab,kw.

2 surgical infection/ or wound dehiscence/ or seroma/ or exp hematoma/ or exp necrosis/ or (surgical site infection* or surgical wound infection* or surgical wound* or surgical incision* or surgical site complication* or surgical site occurrence* or surgical site event* or incision* or prophylactic* or seroma* or necrosis or cellulitis or dehiscence* or hematoma*).ti,ab,kw.

3 1 and 2

CINAHL (Ebsco)

(MH "Negative Pressure Wound Therapy") OR (TI (negative pressure or NPWT or NPT or vacuum assisted closure* or VAC or TNP or TASAP or microdeformational wound therap* or vacuum therap* or closed incision management or surgical incision management or incisional management system* or prevena) OR AB (negative pressure or NPWT or NPT or vacuum assisted closure* or VAC or TNP or TASAP or microdeformational wound therap* or vacuum therap* or closed incision management or surgical incision management or incisional management system* or prevena)) OR (TI topical N6 subatmospheric pressure* OR AB topical N6 subatmospheric pressure*)

AND

((MH "Surgical Wound Infection") OR (MH "Surgical Wound Dehiscence")) OR (MH "Hematoma+") OR (MH "Necrosis+") OR (TI (surgical site infection* or surgical wound infection* or surgical wound* or surgical incision* or surgical site complication* or surgical site occurrence* or surgical site event* or incision* or prophylactic* or seroma* or necrosis or cellulitis or dehiscence* or hematoma*) OR AB (surgical site infection* or surgical wound infection* or surgical wound* or surgical incision* or surgical site complication* or surgical site occurrence* or surgical site event* or incision* or prophylactic* or seroma* or necrosis or cellulitis or dehiscence* or hematoma*)) strategy.

For Peer Review
